# Role of the DEK oncogene in the development of squamous cell carcinoma

**DOI:** 10.1007/s10147-020-01735-5

**Published:** 2020-07-12

**Authors:** Kazuhisa Ishida, Takayuki Nakashima, Toshiyuki Shibata, Akira Hara, Hiroyuki Tomita

**Affiliations:** 1 Department of Tumor Pathology, Gifu University Graduate School of Medicine, 1-1 Yanagido, Gifu, 501-1194 Japan; 2 Department of Oral Maxillofacial Surgery, Gifu University Graduate School of Medicine, Gifu, 501-1194 Japan

**Keywords:** DEK, Oncogene, Squamous cell carcinoma

## Abstract

DEK is a highly conserved nuclear factor that plays an important role in the regulation of multiple cellular processes. *DEK* was discovered to be an oncogene as a fusion with *NUP214* gene, which results in producing DEK-NUP214 proteins, in a subset of patients with acute myeloid leukemia. Subsequently, DEK overexpression was reported in many cancers, thus DEK itself is considered to be an oncoprotein. DEK has been reported to play important roles in the progression of early and late stage squamous cell carcinoma (SCC) and is useful for early diagnosis of the disease. These findings have made DEK an attractive therapeutic target, especially for human papillomavirus (HPV)-associated SCC. However, the mechanism of DEK in SCC remains unclear. In this review, we discuss human *DEK* oncogene-related SCC.

## Introduction

The *DEK* oncogene was initially identified as a target of recurrent t(6;9) translocation, resulting in a fusion with the nuclear pore complex protein-encoding gene *NUP214* in a subset of patients with acute myeloid leukemia (AML) [1, 2]. DEK is a highly conserved nuclear factor and the only member of its protein class, and it has been shown to be preferentially expressed in aggressively proliferating malignant cells. The *DEK* gene is located on chromosome 6p22 − 23 and encodes a 375-amino acid (43 kDa) protein that is abundant in the nucleus where it plays key roles in the architectural control of chromatin assembly [3, 4]. DEK also plays a pivotal role in multiple cellular activities and various cellular metabolic processes, such as maintenance of heterochromatin integrity, transcriptional regulation, mRNA splicing, DNA replication, and DNA repair damage and susceptibility [5].

*DEK* is a Su(var) gene that functions as a positive regulator of heterochromatin, acting through heterochromatin protein 1α (HP1α), which is necessary for the maintenance of heterochromatin integrity [6]. Through its roles in regulating chromatin topology, DEK also regulates various signaling pathways and transcription factors associated with stem cell proliferation, differentiation, and self-renewal [6]. Some reports in humans and Drosophila have demonstrated that DEK inhibits the histone acetyltransferases p300 and p300/CBP-associating factor (PCAF), resulting in histone H3 and H4 hypoacetylation [7, 8]. However, it is unclear whether DEK is essential for heterochromatin establishment and maintaining the balance between heterochromatin, euchromatin, and chromatin. However, it is known that stem cells contain significantly more euchromatin than heterochromatin, and this ratio changes as daughter cells progress through differentiation [9]. Further, cancer cells overexpressing DEK often exhibit heterochromatin instability and marked dysregulation of the epigenome [10].

The frequent upregulation of *DEK* in human malignancies has led to its labeling as an oncogene, and targeted inhibition of DEK has been suggested as a potential strategy for the treatment of different malignancies [11]. DEK has been shown to be upregulated in many malignant conditions, such as AML [1, 2, 11–13], retinoblastoma [14–16], glioblastoma [17], hepatocellular carcinoma [18], oral squamous cell carcinoma (SCC) [19], melanoma [20, 21], and urinary bladder cancer [22–26]. However, the functional mechanisms contributing to the accumulation of DEK in malignant cells are not fully understood.

Interestingly, no mutations have been reported in the coding sequence of human *DEK* gene in tumor tissues [3]. We previously reported that global genomic DNA hypomethylation preferentially suppresses the development of SCC [27]. This led us to hypothesize that SCC might be strongly associated with epigenetic modifications, such as DNA methylation and chromatin remodeling.

In this review, we focused on relationship between the human *DEK* oncogene and SCC. We summarized the current reports implicating *DEK* as a proto-oncogene in SCC and dysplastic disorders and discussed the potential of DEK as a therapeutic target for the selective targeting of cancer cells, especially SCC.

### DEK and cancer stem cells

DEK is not present in quiescent stem cells, although it is expressed in response to environmental cues, inducing an increase in stem and progenitor cells [28]. DEK also helps to maintain the cancer stem cell population, and, at least when present as a fusion protein with CAN in AML, it can induce the transformation of normal stem cells. Thus, abnormalities in the regions of DEK that are necessary for maintaining its normal protein structure might be associated with cancer and cancer stem/progenitor cells [28].

Several reports [29, 30] have indicated that DEK overexpression leads to aberrant chromatin retention, increased mitotic defects, micronuclei formation, and an increased incidence of hypodiploidy and micronuclei formation in cancer cells. These abnormalities led to cellular transformation and carcinogenesis [28, 31, 32]. Dysregulation of DEK is also thought to promote tumorigenesis and sustained proliferation of cancer stem cells [29]. Mechanistically, dysregulation of DEK might promote cell proliferation and survival; modulate signal transduction pathways related to differentiation, migration, and self-renewal; and cause changes in transcription, DNA repair, and replication by altering chromatin organization and resistance to chemotherapy drugs [30].

### DEK expression in different types of squamous cell carcinoma

SCCs can develop in the squamous epithelium of the uterine cervix, lung, esophagus, and other tissues, and several studies have implicated DEK overexpression in SCC (Tables 1 and 2).

**Table 1:** Overexpression of DEK in squamous cell carcinoma (SCC)

Histology	Method	Cases	% cases	Authors (References)
Cervical scc	IHC	≧ 1 (98)	≧ 1 (96.1%)	Wu et al. [39]*
		≧ 2 (82)	≧ 2 (80.4%)	
Lung SCC	IHC		47.90%	Wang et al. [41]**
HNSCC	IHC		100%	Adams et al. [46]***
OSCC	IHC		88%	Nakashima et al. [19]****

*Immunostaining was zero (negative), or < 5% positive cells; one (weak positive), 5–25% positive cells; two (intermediate positive), 26–50% positive cells; three (strong positive), > 50% positive cells. Only nuclear expression was considered as positive staining

**Intensity of DEK nuclear staining was also scored as zero (no staining), one (weak), or two (marked). Percentage scores were assigned as one (1–25%), two (26–50%), three (51–75%), and four (76–100%). The scores were multiplied to give a final score of 0–8, and the total expression of DEK was determined as either negative or low expression (− ; score < 4) or overexpression (+ ; score ≥ 4)

***Number of cells with positive staining was quantified as: three (≥ 90% positive tumor cells), two (10–50% positive tumor cells), or one (< 10% positive tumor cells). The intensity of DEK staining was determined as W (weak), V (variable), or S (strong)

****Number of cells with positive staining scored as: one (0–25%), two (26–50%), three (51–75%), and four (76–100%). The expression of DEK was determined as either negative or low expression (1–2) or overexpression (3–4)

**Table 2:** Overexpression of DEK in precancerous lesions

Tissue	Classification	Method	Cases	% cases	Authors (References)
Cervical lesion	CIN1^a^	IHC	≧ 1 (24)	≧ 1 (85.7%)	Wu et al. [39]*
			≧ 2 (15)	≧ 2 (53.6%)	
	CIN2	IHC	≧ 1 (16)	≧ 1 (94.1%)	Wu et al. [39]*
			≧ 2 (12)	≧ 2 (70.6%)	
	CIN3	IHC	≧ 1 (17)	≧ 1 (89.5%)	Wu et al. [39]*
			≧ 2 (12)	≧ 2 (63.2%)	
Oral lesion	CIS^b^	IHC			Nakashima et al. [19]**

^a^*CIN* cervical intraepithelial neoplasia

^b^*CIS* carcinoma in Situ

*Immunostaining was zero (negative), < 5% positive cells; one (weak positive), 5–25% positive cells; 2 two (intermediate positive), 26–50% positive cells; three (strong positive), > 50% positive cells. Only nuclear expression was considered as positive staining

**Number of cells with positive staining scored as one (0–25%), two (26–50%) three (51–75%) and four (76–100%). The expression of DEK was determined as either negative or low expression (1–2) or overexpression (3–4)

### Uterine cervical cancer and cervical intraepithelial neoplasia (CIN)

Cervical cancer is a common cancer in women worldwide. There are roughly 530,000 new cases and about 275,000 cervical cancer-associated deaths every year [33]. Human papillomavirus (HPV) causes the overwhelming majority of cervical cancer cases [34]. Cervical intraepithelial neoplasia (CIN) is a premalignant stage of cervical SCC characterized by abnormal proliferation of squamous cells in the cervical epithelium [35]. There are three grades of CIN, 1, 2, and 3. In CIN1, the variant squamous cells are localized to the lower layer of the epithelium, and there tends to be minimal nuclear abnormalities and mitotic features. In CIN2, cellular dysplasia is confined to the lower half of the epithelium, and there also tends to be more pronounced nuclear changes and mitotic features. In CIN3, cellular dysplasia and cell polarity are present in all layers or only the superficial layers of the epithelium. In addition, nuclear abnormalities and mitotic features, with a general loss of cell polarity, can be observed throughout the epithelium [36].

DEK is largely localized in the nucleus. Soares et al. [35] demonstrated, using immunohistochemistry, that DEK was overexpressed in the nuclei of uterine cervical cancer cells, including SCC cells (Table 1). DEK protein was expressed in the nuclei of only 2–3 layers from the basal layer of the non-neoplastic cervical epithelia, and the density of DEK-positive cells was lesser than that in the cancer cells [37]. Compared to normal cervical epithelial cells, DEK protein expression was higher in CIN1, CIN2, and CIN3 cells, as suggested by the strong positive signals [37] (Table 2). Moreover, DEK protein was strongly expressed in SCC (score ≧ 1, 96.1%, 98/102 cases; score ≧ 2, 80.4%, 82/102 cases). Only nuclear expression was considered as positive staining in cases. The immunostaining was semiquantitatively scored as zero (score 0), 0–5% positive cells; one (score 1), 5–25% positive cells; two (score 2), 26–50% positive cells; and three (score 3), > 50% positive cells [37]. These results suggest that DEK plays an important role in the early stage of cervical cancer.

### Squamous cell carcinoma of the lung

Non-small cell lung cancer (NSCLC) is the most common type of lung cancer, and it is further classified into SCC, adenocarcinoma, large cell carcinoma, and non-small cell carcinoma. SCC of the lung accounts for 20–30% of all NSCLCs [38].

Wang et al. [39] performed an immunohistochemical analysis of 112 NSCLC and 38 normal lung tissue samples and found that DEK protein expression was higher in the lung cancer tissues than that in the normal lung tissues. In addition, while DEK staining was negative in normal bronchial epithelial cells, DEK was overexpressed in tumor cells, mainly in the nuclear compartments. DEK-positive expression was detected in 47.9% (23/48) of SCC cases [39]. Xin et al. [40] also reported that DEK expression was significantly correlated with poor differentiation and advanced clinical staging. NSCLC patients with DEK-expressing tumors had a lower disease-free survival rate and overall survival rate than patients without DEK expression. In early stage NSCLC, patients with DEK expression had lower disease-free and overall survival rates than patients without DEK expression [31, 40]. DEK expression was found to be significantly higher in lung adenocarcinoma than in SCC. These data suggest that DEK may play an important role in the progression of NSCLC and may be an important biomarker for evaluating the prognosis of lung cancer.

### Squamous cell carcinoma of the head and neck

Head and neck cancer is a broad entity that encompasses epithelial malignancies arising from the paranasal sinuses, nasal cavity, oral cavity, pharynx, and larynx. Head and neck squamous cell carcinoma (HNSCC) is the sixth most common human cancer [41], with nearly 600,000 new diagnosed cases and approximately 350,000 deaths each year worldwide. Infection with high-risk HPV types has been identified as a novel risk factor for a subset of HNSCCs, particularly oropharyngeal cancer [42].

Approximately one-third of all HNSCC cases are oral squamous cell carcinoma (OSCC). HNSCC has been shown to develop through a series of dysplastic changes before progressing to invasive cancer [41]. Precancerous lesions in the keratinizing epithelium of the upper aerodigestive tract, include leukoplakia, erythroplakia, and mixed leukoerythroplakia (hyperplastic epithelial lesions). These clinically defined lesions have been reported to carry a higher risk of transformation into SCC as compared to normal mucosa [43].

DEK expression has been implicated in HNSCC. Adams et al. [44] performed an immunohistochemical analysis of DEK expression in human head and neck carcinoma tissues. They assessed the intensity of DEK protein expression and the proportion of DEK-positive tumor cells relative to the adjacent normal tissue. The analysis showed that DEK was expressed in all tested tumors [44]. Similarly, we performed an immunohistochemical analysis of DEK expression in 34 human OSCC tissue samples and normal oral tissues [19]. In the normal tissues, we found that DEK protein was only expressed in the nuclei of the basal layers. DEK protein expression was higher in OSCC tissues than in the normal tissues, and the percentage of positive cells was > 50% in almost all samples (30/34 cases) [19]. These studies implicate DEK overexpression in the progression of head and neck cancer.

Recently, two DEK-overexpressing murine models have been reported [19, 45]. Matrka et al. [45] established a tetracycline-inducible DEK transgenic mouse model to investigate whether DEK contributes to carcinogenesis in vivo. In the transgenic mouse model, DEK was overexpressed in specific tissues, and expression was inhibited by doxycycline. These mice were exposed to the chemical carcinogen 4NQO to induce cancer in the oral cavity and esophagus. The results showed that DEK overexpression increased the overall incidence of esophageal SCC as well as cellular proliferation in adjacent non-tumor tissues. SCC has been reported to arise from keratinocytes in the squamous epithelium, and overexpression of DEK has been shown to promote cell proliferation and transformation and inhibit apoptosis [11, 46–48]. Matrka et al. [45] concluded that cell differentiation, senescence, and DEK overexpression specifically targeting the basal keratinocytes can promote the proliferation of cells and the development of SCC in vivo.

Nakashima et al. [19] established a similar doxycycline (DOX)-inducible DEK mouse model (referred to as *iDEK* mouse). They also established a squamous cell-specific DOX-inducible DEK mouse model (referred to as iDEK-e mouse). However, the iDEK and iDEK-e mice did not show any changes in the oral mucosa following administration of DOX and 4NQO. However, in a microarray analysis, DEK overexpression was found to be mediated by the upregulation of DNA replication and cell cycle-related genes, particularly those involved in the G1/S transition. Although there are some differences between the two studies (Table 3), they yielded markedly contrasting results. DEK overexpression upregulates genes involved in the cell cycle and cell replication, but the functions of DEK are very complicated [7, 49]. For this reason, it is important to consider the potential roles of extracellular DEK in maintaining the tumor microenvironment and regulating immune functions. The functions of both intracellular and extracellular DEK need to be studied further to develop targeted therapies.

**Table 3:** The differences between two Tetracycline-inducible DEK transgenic mice

	Targeted	4NQO	4NQO	DOX	Mouse
		Exposure	Concentration	Exposure	
Matrka et al. [Reference 47]	Basal epithelial cells*	16 weeks	10 μg/ml	45 weeks	FVB/N
Nakashima et al. [Reference 19]	Basal epithelial cells** or Ubiquitous cells***	28 weeks	20 μg/ml	4 weeks	C57BL/6

*Basal epithelial cells (Krt5 promoter-dependent)

**Basal epithelial cells (Krt14 promoter-dependent)

***Ubiquitous cells (Rosa26 promoter-dependent)

Cancer immunotherapy is used in many cancer patients as a major method for cancer treatment. In the human immune system, the tumor suppression effect is exhibited by the cooperation of immunostimulatory neoantigens and T cell-mediated cytotoxicity [50, 51]. Yang et al. [52] focus on this mechanism, have experimented about immune-check inhibitors and *DEK* gene with regards to SCC treatments. According to them, the *DEK–AFF2* fusion gene was the likely driver event in head and neck squamous cell carcinoma, neoantigens derived from the *DEK-AFF2* fusion may induce an immunostimulatory T cell response. And then, expression of the DEK–AFF2 protein induced a cytotoxic T cell response against SCC-9 cells. We think that further analysis of this mechanism can improve immune checkpoint inhibitors for head and neck cancer.

### HPV infection and DEK in squamous cell carcinoma

The role of HPV-induced carcinogenesis has been extensively studied in cervical cancer, the most widely accepted HPV-related malignancy. Almost all cervical cancers are initiated by infection with high-risk HPV [53]. Importantly, DEK overexpression has been shown to be induced by high-risk, but not low-risk, HPV E7 protein in a retinoblastoma protein (Rb) function-dependent manner. It has been reported that DEK overexpression inhibits apoptosis in HeLa cervical cancer cells, and it can also inhibit p53 transcriptional activity [46]. DEK overexpression has been implicated in the inhibition of cellular senescence, indicating that DEK plays a very important role in the progression of cervical cancer [12, 54]. Wu et al. [37] showed that DEK protein was highly expressed in both HPV-positive and -negative cervical cancer cells as well as precancerous lesions. Furthermore, the authors [37] detected DEK expression in both HPV-positive and -negative cervical cancer cell lines, irrespective of HPV status [17, 54]. This suggests that it might be important to investigate the relationship between HPV infection and DEK protein expression in cervical cancers, and further studies are needed to explore the mechanisms of *DEK* upregulation in the progression of cervical cancer.

The causative relationship between high-risk HPV and OSCC is well established, and HPV-associated OSCC is a distinct entity from tobacco-associated OSCC. Virus-associated cancers continuously express the HPV E6 and E7 viral oncogenes even in the advanced stages of the disease. Repression of viral oncogene expression can prevent the growth and survival of cancer cells [55]. It was recently shown that a subset of head and neck cancers is HPV positive. Interestingly, this subset is biologically distinct and more sensitive to chemoradiation therapies, although the underlying mechanism is unclear [48]. Similar to cervical cancer, *DEK* is upregulated in numerous head and neck cancers, regardless of HPV status [48]. Developing novel treatment strategies targeting potential oncogenic candidates, such as DEK, is of paramount importance to improve therapeutic outcomes in patients with HPV-related SCC. However, the potential of DEK as a therapeutic target remains to be explored.

## Conclusion

In conclusion, DEK protein is overexpressed in human SCCs in multiple organs, and it plays an active role in tumor initiation and maintenance. Although there are still many open questions regarding the regulation and function of this protein, elevated DEK protein expression may be useful as a novel prognostic factor for SCC patients, and DEK may be a novel therapeutic target for human SCC.
